# Non-exercise activity thermogenesis in the workplace: The office is on fire

**DOI:** 10.3389/fpubh.2022.1024856

**Published:** 2022-10-28

**Authors:** Alex Rizzato, Giuseppe Marcolin, Antonio Paoli

**Affiliations:** Department of Biomedical Sciences, University of Padova, Padua, Italy

**Keywords:** energy expenditure, sedentary behavior, workplace, sitting, physical activity

## Abstract

From the second half of the previous century, there has been a shift toward occupations largely composed of desk-based behaviors. This, inevitably, has led to a workload reduction and a consequent lower energy expenditure. On this point, small increments of the non-exercise activity thermogenesis (NEAT) could be the rationale to reach health benefits over a prolonged period. Different published researches suggest solutions to reverse sitting time and new alternative workstations have been thought to increase total physical activity. Therefore, the purpose of this narrative review is to summarize the current state of the research regarding the “NEAT approach” to weight-gain prevention in work environments. This review analyzes the main evidence regarding new alternative workstations such as standing, walking workstations, seated pedal, and gymnastic balls to replace a standard office chair.

## Introduction

### Historical background and epidemiological frame

Over the past 50 years, technological development and the making of ever-new labor-saving devices have reduced physical activity and, consequently, energy expenditure (EE) across many different domestic and working settings (1). The term sedentary etymologically refers to “remaining in one place” from Middle French *sédentaire* (1590s) and directly from Latin *sedentarius* “sitting, remaining in one place.” Later recorded in the 1660s, Proto-Indo-European referred to persons, in the sense of “not in the habit of exercise.” Nowadays, it refers to a specific group of activities that involves low levels of EE in the range of 1.0–1.5 Metabolic Equivalent of Task or MET (1 MET is defined as 3.5 mlO_2_/Kg/min): for example, sitting during transfers (i.e., by train or car), tasks performed while working, and for leisure or in the domestic location (2). A growing amount of evidence contended that the increased tendency to a sedentary lifestyle plays a main role in the rise of multiple chronic diseases, including cardiovascular disease, Type 2 diabetes (3), and overweight and obesity (4). Regarding the latter, despite the rising problem awareness, the obesity epidemic is constantly growing and obesity rates are increasing worldwide. In 2016, more than 1.9 billion adults, 18 years and older, were overweight. Of these, over 650 million were obese. About 39% of adults aged 18 years and over were overweight in 2016 and 13% were obese (5). As recalled, since a sedentary lifestyle represents one of the main risk factors for developing chronic diseases, disablement, and frailty, reducing the time spent in sedentary activities is a population-wide goal for positive health outcomes. Twenty-five years ago, Prentice et al. (6) published a study about the secular trends in diet and physical activity and obesity in Britain after retrospectively analyzing data from 1950 to 1990. Evidence suggested that changes in the prevalence of obesity were not related to changes in total energy or fat intake. Conversely, indirect measures of physical inactivity (i.e., car ownership and hours of television watching) appeared to be more closely related to a change in body weight. More recently, an epidemiological study differentiated sedentary sitting time alone from sedentary TV-viewing time. High levels of moderate-intensity physical activity (i.e., about 60–75 min per day) seemed able to eliminate the increased death risk related to the sitting time alone, having instead a little influence on the increased risk of death related to TV-viewing time (7). Ekelund et al. (7) hypothesized two possible explanations regarding this association. The first theory is that TV-viewing mainly happens after dinner, and postprandial sedentary time may be detrimental to glucose and lipid metabolism. Moreover, behaviorally, TV-viewing is frequently accompanied by snacking or other eating habits possibly influenced by TV advertising. In addition to morbidity and early mortality, a sedentary lifestyle is “guilty” of a considerable economic burden. A world global analysis revealed that physical inactivity cost for healthcare systems was $53.8 billion in 2013, of which $31.2 billion was borne by the public sector, $12.9 billion by the private sector, and $9.7 billion by households (8). The recent COVID-19 pandemic has increased sedentary behaviors during the imposed lockdown periods across several populations, including children and patients with a variety of medical conditions (9). Thus, multiple interventions targeting sedentary behaviors have been studied; for instance, Gardiner et al. studied the efficacy of a face-to-face goal-setting consultation and one individually tailored mailing providing feedback on accelerometer-derived sedentary time in a group of older adults. They found a decreased sedentary time (– 3.2%), increased breaks in sedentary time per day (i.e., four), and an increased level of light (2.2%) to moderate to vigorous (1.0%) physical activity (10). Moreover, the amount of sitting time, prolonged or interrupted, is significantly associated with cardiovascular disease risk in adults over age 45 years (11). Larsen et al. showed that interrupting the sitting time every 20 min positively influenced systolic blood pressure, such as reducing all-cause mortality risk by 3–4% (11). In addition, regular breaks during prolonged sitting periods lowered postprandial glycemia in middle-aged adults without metabolic impairment (12). Indeed, humans expend energy also having routinary postures and performing daily-living movements (e.g., standing, walking, stair climbing, and many others). Thus, some daily non-exercise activities, also alternated with prolonged sitting, could considerably contribute to an increase in total daily energy expenditure. In this regard, the “Compendium of Physical Activities” continued to accumulate and categorize published reports of the EE in MET associated with different physical activities (13, 14).

### Humans: Evolutionary active animals

**Figure 1:**
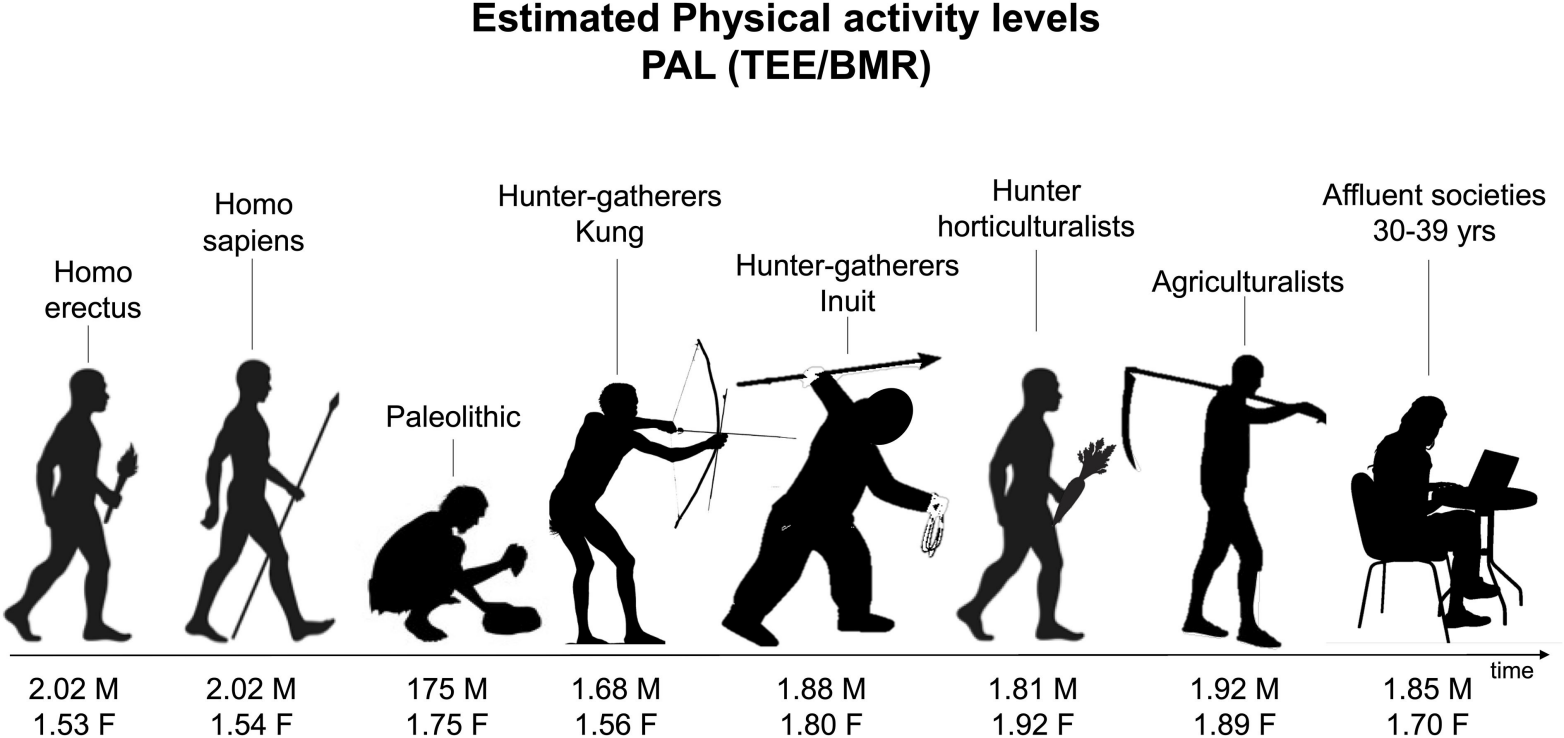
Estimated physical activity levels (PALs) for ancestors and modern populations. PAL = TEE/BMR (total energy expenditure/basal metabolic rate), see for details, Malina and Little (17).

Although sedentary behaviors encompass large sections of the population, evolutionary biology suggested that humans are not physiologically adapted to periods of prolonged inactivity. In industrialized countries, activities that require daily locomotion to man are often very low. Thus, nowadays, human energy expenditure is reasonably lower than in our Paleolithic ancestors (15). This evolutionary theory could also account for the increase in obesity prevalence rates worldwide. Hayes et al. found that the physical activity levels of humans living in the modern environment were much lower than that observed in free-ranging mammals, used as a model for primitive humans (16). Unfortunately, data on energy expenditure from physical activity in prior times are lacking and it is possible to lend support to this hypothesis only through estimations. Malina et al. (17) presented an estimated summary of physical activity levels (PALs) along our evolutionary past (Figure 1). Humans are biologically equipped to be physically active, however, cultural development allowed sedentary chances. Moreover, not only periods of prolonged inactivity but also how this inactivity time is spent is crucial in the burden of the sedentary lifestyle. Reduced energy expenditure deriving from decreased muscle activity is responsible for the increased health risk due to chair-seated postures (18). In this regard, Raichlen et al. studying the non-ambulatory time, observed that, with respect to the industrialized population, the Hazda (an African hunter-gatherer population) spent their resting time in “active” rest postures (19). Moreover, the authors showed that these postures require significantly higher energy levels for lower-limb muscle activation than chair sitting calculated through estimation in the percentage of walking (19). For instance, in the assisted squat posture, muscle activation of the soleus (right: 10.831; left: 10.883) was significantly higher compared to chair sitting (4.943). Again, the full squatting posture elicited higher levels of muscle activity compared with chair sitting for soleus (left: 8.395; right: 15.086 vs. left: 4.943; right: 5.754), vastus lateralis (left: 14.616; right: 29.800 vs. left: 5.927; right: 10.508), and tibialis anterior (33.239 vs. 3.742). Thus, despite the sedentary time in the Hadza population is not lower than in industrialized people, Hadzas showed low levels of biomarkers related to an increased risk of cardiovascular diseases (19).

### NEAT, an important part of total daily energy expenditure

Levine (20) defined non-exercise activity thermogenesis (NEAT) as the physical activities other than volitional exercises, such as the activities of daily living, fidgeting, spontaneous muscle contraction, and maintaining posture when not recumbent. Together with resting energy expenditure, postprandial thermogenesis, and physical activity thermogenesis, NEAT composes the total daily EE. NEAT differentiates from physical activity and is defined as “any bodily movement produced by skeletal muscles that resulting in EE above the resting level,” usually over 1.6 MET (21, 22). To better comprehend NEAT and its role in individuals with obesity, we can divide it into posture-related (e.g., standing, sitting, and lying) and movement-related thermogenesis (e.g., walking, occupation, and leisure time). In sedentary adults, EE deriving from NEAT helped to counteract weight gain during controlled overfeeding experiments (23). Von Loeffelholz (24) showed that NEAT could widely vary up to 2,000 kcal·day^−1^ between two individuals of similar size, lean body mass, and gender. The authors explained these differences with the interactions of several biological and environmental factors; indeed, it was given great importance to people's different occupations and leisure-time events. In a sedentary job, NEAT could range at a maximum of 700 kcal·day^−1^, as average (24). These data confirmed those from Ravussin et al. (25), who used a human respiratory chamber to determine rates of EE over 24 h. They found that variability in the degree of spontaneous physical activity (range 100–800 kcal/d) could account for a large portion of daily EE (25). Among spontaneous movements or behaviors promoting NEAT, fidgeting has also been associated with weight loss across long periods of time (26). Fidgeting is defined as making continuous, small movements, typically with hands or feet, in a nervous or restless way, that is unnecessary to the ongoing task (27). These movements can occur while sitting or standing. An interesting study by Hagger-Johnson et al. (27) retrospectively examined the association between sitting time and mortality in almost 13,000 women from 1999 to 2002. They found that fidgeting minimizes the association between sitting time and mortality in the medium (5–6 h) and the high (7–17 h) fidgeting groups. Given the above, the employment of simple behaviors might contrast the negative consequences of time spent sitting, independently from the level of physical activity, hence, fidgeting appears to be sufficient to influence daily energy balance (28) with long-term health benefits, even to sedentary individuals. However, despite fidgeting representing a topic of interest for many researchers, measuring it with reliable markers is still an issue. When studying spontaneous physical activity, combining information from self-report and accelerometers (29, 30) together with the proper assessment of the subject's sitting position and specific limb movements appears to be necessary.

Levine suggested that the environmental factors promoting sedentary behavior affect differently obese and lean individuals; specifically, if subjects with obesity adopted the NEAT-enhanced behavior typical of their lean counterparts, they could expend an additional 350 kcal per day. With an unchanged energy intake, this could result in a weight loss of ~15 kg over a year (31). On this point, small increments of NEAT could be the rationale to reach health benefits over a prolonged period. However, adults spend most of their days working (about one-third), and the work is surely becoming more sedentary (32). Understanding how much the occupational sedentary lifestyle count in reducing the total amount of time spent actively is the first step to directly program interventions in workplaces. Therefore, the purpose of this narrative review is to summarize the current state of the research regarding the “NEAT approach” to weight-gain prevention in work environments. Moreover, analyzing the variety of research strategies to increase NEAT at work, the review aims to point out questions, gaps, and openings.

## Sedentary behaviors at work

From the second half of the previous century, there has been a shift toward occupations largely composed of desk-based behaviors. In the 1950s, Morris et al. already stated, “men in physically active jobs have a lower incidence of coronary (ischaemic) heart disease in middle-age than men in physically inactive jobs” (33). This trend has also been associated with population-level weight gain (34). Indeed, typical adult weight gain results from a daily positive energy balance of 15–50 kcal/day (35). This low amount of daily energy intake excess might not appear of clinical relevance and thus, its relevance for weight gain may be underestimated. The cumulative effect of very small daily weight gains is very likely to be a substantial contributor to the overall increase in body weight that frequently occurs during adulthood (36). Moreover, long periods of desk-based behavior have been linked to increased pain and musculoskeletal disorders. Specifically, Jensen et al. showed that jobs characterized by the highest level of repetitiveness (i.e., call center and data entry works) are associated with an increased rate of discomfort in the neck, shoulders, and upper extremities (37). In the scientific literature, several methods have been used to evaluate the level of physical activity at work, such as self-report (38), surveys (39), questionnaires (40), and motion sensors (41). For instance, Thorp et al. (42) conducted a study quantifying the sedentary working time using accelerometers in 193 employees. They concluded that working hours were mostly spent sedentary and that the working days were more sedentary and had less light-intensity activity than non-working days. However, a review proposed by Castillo-Retamal complained that there was a substantial inconsistency in assessing physical activity at work and that none of the studies considered the validity or reliability of these measures (22).

## Strategies to increase NEAT at work

Technological development has addressed office ergonomics and, more in general, the environmental design toward a constant effort saving. This, inevitably, has led to a workload reduction and a consequent lower EE (43). Recently, different published researches suggest solutions to reverse increasing sitting time and encourage daily movement in the working scenario to reduce the risks connected to a sedentary lifestyle (20, 44, 45). New alternative workstations have been thought to increase total physical activity in sedentary workers and improve body composition (i.e., decreasing body fat) (46). The additional EE favored by alternative workstations should increase the NEAT and should be bearable for prolonged periods. However, dynamic workstations may carry limitations due to mental distraction that could affect work productivity or safety (45). For this reason, the design and engineering of alternative workstations should guarantee the normal execution of working tasks, indeed, workers risk finding themselves sitting on the fence between their NEAT increase and their working yield. For this reason, any modifications of the working scenario must carefully weigh the advantages and disadvantages of its ecological application. Following, we summarize the different methods to endorse EE in the working scenario. We reviewed the main evidence regarding new alternative workstations such as standing, walking workstations, seated pedal, and gymnastic balls to replace a standard office chair.

### Standing workstation

It is well known that posture changes have chronic and acute relapses in many physiological variables such as metabolic rate, anti-gravitational muscle tone, and cardio-circulatory indexes (47–51). However, the actual query is when these changes, even significant, become relevant in terms of energy balance for weight gain prevention. On this topic, few controversial responses were observed. Indeed, some evidence demonstrated a greater EE while performing clerical work standing with respect to sitting (52). Speck et al. hypothesized that standing could increase the total daily EE over sitting by 384 kcal (i.e., 1,104 vs. 720 kcal). However, their experimental findings, using indirect calorimetry, demonstrated that full-time (8 h) standing workers did not gain the EE equivalent to an hour of daily moderate physical activity (53), while recommendations state that physical activity levels to prevent weight gain must be ≥1.6 times the basal metabolic rate (54). Again, Tudor-Locke et al. (55) strengthened the assumption that replacing sitting behaviors only with standing appears to be insufficient in terms of EE. Even though the focus should last on EE, other potential health benefits of standing than sitting position need to be acknowledged. For instance, Beers et al. found a significantly higher heart rate in standing than in seated posture during a word processing task (52). Thus, the standing posture could partially counterbalance the low physical activity associated with the seated position. An increased EE in the standing position due to higher muscle activation was also supposed. Available data showed higher muscle activation in the lumbopelvic region when maintaining erect postures compared to passive seated postures (56). Indeed, we demonstrated that anti-gravitational muscle tone increment in the standing posture is a major determinant of metabolic rate changes (57). In addition, Tikkanen et al. showed a higher thigh muscle activation during standing compared to sitting posture (58). However, possible disadvantages in maintaining a prolonged standing posture can occur. Epidemiological studies suggested that prolonged standing might be related to health problems such as venous insufficiency (59), decreased cognition or discomfort (60), and back pain (61). Occupational standing has been associated with elevated low back pain. Indeed, between 40 and 70% of the population who never had a low back injury are categorized as developing pain when exposed to a bout of static prolonged standing using self-reports (62, 63). On the other side, standing for >50% of a workday did not affect the pulse wave velocity of standing workers more than their seated counterparts, showing non-adverse effects on their arterial stiffness (64). Finally, subjective feeling of comfort, fatigue, and liking experienced during the standing posture is not a secondary topic (52) that can easily affect workers' productivity. Nowadays, there are no univocal guidelines to modulate sitting and standing times, as every person has dissimilar necessities and functional impairments (65). Thus, if sitting time can be harmful, standing time is not fully harmless.

### Walking workstation

Among the activities recommended to increase NEAT, walking is one of the most feasible for almost all subjects. Thus, behavioral engineering and ergonomics studied different methods to increase walking, and consequently EE, in the workplace. For instance, many companies decided to remove e-mail or telephone for the correspondence between colleagues to stimulate walking or introduced a 10-min walking break during working hours. Straker et al. (43) well summarized the proposed solutions in three categories: equipment changes (e.g., walking to the printer on the second floor), task changes (e.g., workers do different working tasks in rotation), and organizational changes (e.g., information and sensitization activities for physical activity). Together with these NEAT-increasing solutions, walking workstations have also been developed through a treadmill placement at the workers' desk. The walking workstation consists of a setup that allows for walking slowly on a treadmill while working at a raised desk. In the late eighties, Edelson et al. already recommended walking on a treadmill to increase physical activity at work without a concurrent decrement in working performance (66). If activities with very low workloads, such as the aforementioned fidgeting, can increase by 20–40% EE over resting levels (28), walking can multiply basal EE (67). Indeed, Levine et al. estimated that walking at 1.6 km/h (e.g., quiet walking for shopping) doubles EE and that intentional walking at 3.2 to 4.8 km/h led to a doubling or tripling EE (68). Moreover, walking at 6.4 km/h has a MET level 5 times greater than sitting at rest (69). However, this topic deserves to be analyzed in a work context. Longitudinal studies investigated the long-term effects of a treadmill-desk program and showed a positive effect on anthropometry, body composition, blood lipids, and metabolic indexes (70–72). Walking workstations instead of standing workstations led to the greatest improvement in different physiological outcomes, including postprandial glucose and HDL cholesterol (73).

Besides, walking at a very slow speed of 1.7 km/h on a treadmill while working increased heart rate up to 15 bpm (74) and EE up to 119 kcal/h, as average (75) above the seated working condition. The reported walking EE is almost 2.7 times above the estimated EE in seated work (averaged at 72 kcal/h). For instance, full-time employment of treadmill workstations could utopianly lead to an EE of 4,800 kcal/day obviously without considering problems of tolerance, pleasure, or discomfort (55). It has been hypothesized that the daily use of a treadmill workstation for 2.5 h/day in subjects with obesity may lead to an estimated weight loss of 20 to 30 kg/year (75). However, Levine et al. (75) only prospectively estimated NEAT for weight loss starting from controlled research of short duration. Although the total daily amount of physical activity is positively affected by treadmill desks compared to standard chairs, the possible altered working performance is noteworthy to discuss. Research investigating this issue showed inconsistent results. More in detail, Thompson et al. (76) demonstrated that subjects using a walking workstation employed longer time in working tasks compared to sitting, while the accuracy in completing them remained unchanged. Conversely, other studies showed that exercising at moderate intensity had beneficial effects on task-speed solving, but not in its accuracy (77). Although the ideal walking velocity for letting the working performance unaffected is still under debate, results suggested 2.25 km/h for word processing tasks (78). Moreover, a systematic review suggested that a self-selected pace between 1.6 and 3.2 km/h is ideal for optimizing typing and mouse performance (73).

One of the issues related to walking workstations is that the continuous changes in the surrounding environments, acting forces, and sensory inputs could lead to a higher cognitive-motor interference due to increased information processing very similar to what occurs under dual-task conditions (79–81). Accordingly, Larson et al. (82) found no influence on executive and cognitive functions while working on a walking treadmill, even though fine motor skills and learning were negatively affected. These results were partially confirmed by Podrekar et al. (83) who showed a decreased working performance during walking activities, but not a worsening of cognitive functions (e.g., attention, learning, and memory). Thus, the hypothesis was that a higher familiarity with the device and its long-term employment and practice could have improved the worker's performance. Finally, since the several positive effects that walking workstations have demonstrated to produce on EE, additional studies are necessary to deepen the possible worsening of working performance and determine optimal walking speed. Moreover, although there is a relative abundance of short-term evidence, longitudinal studies appear essential to strengthen the observed outcomes over the long-term application of walking workstations.

### Seated pedal workstation

Standing or walking workstations force the employees to work in an unusual setting. Thus, an alternative method to defeat working sedentary behavior is to transform sitting into “active sitting” (84). The rationale is to promote NEAT while remaining in the most habitual seated position, averting an eventual decrease in working performance more likely in standing or walking workstations. Seated pedal workstations are easily manageable by workers who can alternate active pedaling to standard sitting, simply stopping leg movements (55). Peterman et al. (85) studied passive cycling (i.e., external motor moved subjects' legs) by considering how pedaling cadence (at 60 and 90 rpm) can influence EE and heart rate. During two-leg passive cycling, EE rates were significantly greater than rest for both 60 rpm (28%) and 90 rpm (49%). Heart rate showed no significant differences. Moreover, Carr et al. (86) showed that working at a seated active pedal workstation significantly increased EE (53.4%), heart rate (12%), and muscle activation of the biceps femoris (42.1%) and vastus lateralis (59.8%) over the sedentary workstation. The experimental trials were conducted at a pedaling cadence of 45 rpm, comparable to 2.25 km/h. Moreover, Horswill et al. (45) studied the HOVR device, a pendulum with two discs at the end that allows leg movement under the desk. When workers performed leg movements there was an increase in metabolic rate (by 17.6% and 7%) compared to sitting and standing, respectively. Studying the same HOVR device, Koepp et al. (84) found a significant increase (18%) in EE while using the under-the-table apparatus compared to the standard chair. However, the observed changes were much lower compared to a 1.6 km/h walking. Levine et al. found an increased EE in workers using an under-desk device for leg movement (98 ± 42 kcal/h) and a chair promoting fidgeting (89 ± 40 kcal/h) compared to the use of a standard chair (76 ± 31 kcal/h) (87).

As before, the employment of pedal workstations cannot disregard the workers' tolerance and productivity. There was agreement among decrement of some finer motor tasks (i.e., mouse pointing, click time, and typing) using a pedal workstation compared to the standard chair. Besides, reported decrements in seated conditions (with or without pedaling) were surely lower than observed while walking (74). However, results were controversial on whether cognitive functions were altered (74) or not (86). Users' liking and perceptions on the choice of the most suitable workstation has certain effects on their working performance. Tardif et al. (88) tested users' experience through a questionnaire on using a pedal or a standing desk. They found a greater appreciation of the pedal desk over standing for its effective, useful, functional, convenient, and comfortable dimensions. During a standard 8-h working day, 97.6% of subjects reported that their typing proficiency could not be influenced by a 4-h employment of the pedal desk (89). Moreover, besides working productivity, the rates of compliance deserve attention. Indeed, a high number of hours and days of use are needed to improve health over long periods (90). On this point, even though workers reported the pedaling workstation as a feasible intervention, Carr et al. showed actual compliance of 61% over 20 days and 37.7% over 84 days (91). These studies suggest that workers may have used the devices primarily during work breaks and that further environmental modifications are necessary to encourage long-term use. Overall, findings from scientific literature globally suggested that seated pedal workstations offered a good balance between increased EE and affection for working performance. Indeed, it represents a tool to increase daily levels of NEAT, with a keen eye on work quality and workers' appreciation.

### Gymnastic ball workstation

The employment of unstable devices such as gymnastic balls is a popular practice in athletic professional (92), recreational (93), and rehabilitation (94, 95) contexts. Several information channels frequently suggest gymnastic ball sitting (at work, home, libraries, and in many other environments), not always with scientific awareness (96). Indeed, gymnastic balls with respect to conventional chairs do not provide a stable base of support and thus may require a higher commitment to maintaining the body posture on top (97). Subjects are constantly constrained to find balance adjustments to maintain their posture (98). Thus, to preserve an adequate upright posture while sitting on the gymnastic ball, subjects should increase muscles' activation and experience increased heart rate, with a consequent higher metabolic rate (56, 99). In this regard, Haller (99) demonstrated that EE was significantly higher (5.6%) while sitting on a gymnastic ball than in a standard chair. These findings are very similar to those in a later study that found a higher EE (6%) when working on the gymnastic ball than while sitting on the standard chair. EE registered in subjects seated on the gymnastic ball was also very similar to that observed during the standing position (52). These EE increments produced an estimated additional net of 32 kcal/day when calculated over a full-time working day (55). As aforementioned, even though small, this extra amount of EE could successfully influence weight gain prevention (35). Although gymnastic ball application needs further insights to deepen its role on EE, other aspects of “active sitting” require to be acknowledged. For instance, gymnastic ball employment in workplaces could improve posture and muscle activation (100). However, controversial results can be found in the scientific literature.

Gregory et al. investigated trunk muscle activation and posture, comparing a standard office chair to a gymnastic ball. Among the registered muscles (i.e., thoracic and lumbar erector spinae, rectus abdominis, and external oblique), only the thoracic erector spinae was found to increase muscle activation (101). Similarly, Kingma et al. (102) found greater trunk motion (33%) and variation in lumbar electromyography activity (66%) in subjects seated on a gymnastic ball compared to an office chair. Conversely, other authors showed no difference in trunk muscle activation when users sat on a gymnastic ball compared to a stable stool (103). Even though some authors showed an increased self-perceived posture (100), long-term use of gymnastic balls could be unproductive if accompanied by discomfort (101–103). Other researchers suggested that trunk muscle strength could positively influence the experienced discomfort, often related to low back pain (104, 105). However, it is hard to infer if an increase in muscle strength could be due to the working employment of the gymnastic ball. Finally, workers can easily adopt gymnastic balls to obtain small behavioral changes and reduce sedentary negative behaviors. However, understanding whether the advantages of using a gymnastic ball may offset the disadvantages is still an open question, especially over long periods.

## Conclusion

**Figure 2:**
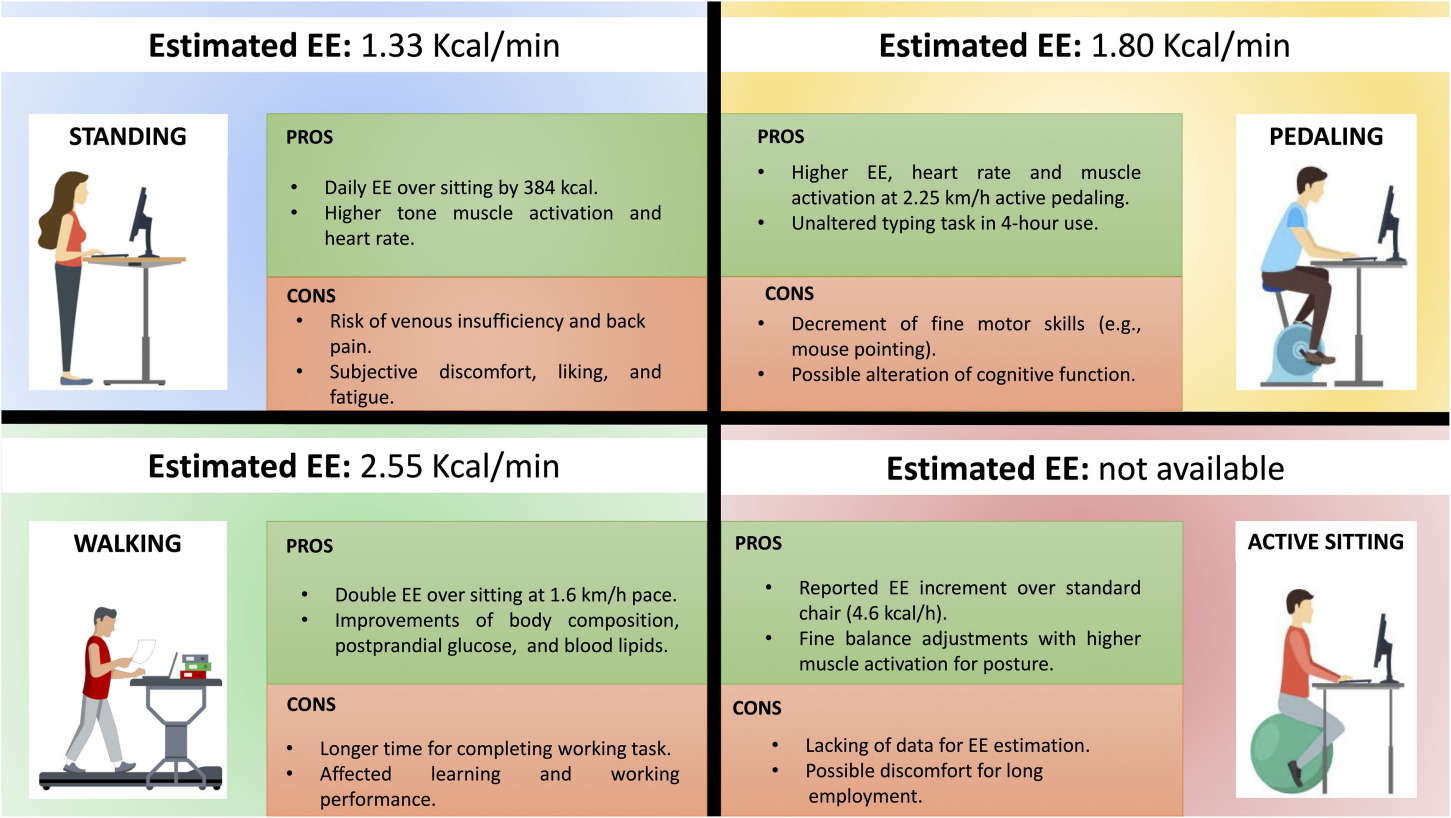
Alternative workstations. Summary of the main pros and cons of the standing, walking, pedaling, and gymball sitting workstations.

The NEAT approach in the workplace could contribute to consciously increasing activity in sedentary workers. Surely, structured exercise programs and an out-of-work active lifestyle represent the best solutions to counteract epidemic obesity. However, many people scarcely spent their leisure time doing physical activity due to other competing personal, domestic, and civic obligations. Indeed, as the working day takes up a large amount of the daytime, the application of alternative workstations will assist in the maintenance of a healthy weight. Theoretical frameworks suggested that NEAT is impacted by the environment (26). As such, using alternative workstations in an 8-h working day might be enough to slow down epidemic obesity. Moreover, Hill et al. showed that the median of the distribution of estimated energy accumulation is 15 kcal/day, and 90% of the population showed a surplus of 50 or fewer kcal/day. This means that an intervention that aims at reducing energy excess by 50 kcal/day could offset weight gain in about 90% of the population (106). Table 1 summarizes the EE (kcal/min) of the above-analyzed alternative workstations. As an assumption, considering that 1 kg of fat is equivalent to ~7,000 kcals, the kilograms of fat consumed over a year are reported for each workstation. Thus, an increase in EE (Table 1) was estimated for standing (~1.1 kg/yr), seated pedal (~9 kg/yr), and walking (~21.5 kg/yr) workstations over the standard seated position. We calculated these values through simplistic estimations considering 8 h of a working day, 22 working days in a month, and 11 working months in a year. Unfortunately, the only evidence about EE on the gymnastic ball workstation (52) makes hard the comparison with the other alternative workstations. As a result, considering the potential benefits associated with unstable devices (56, 99), this lack claims updated scientific evidence. The reported increment (4.1 Kcal/h) over the standard seated position is not sufficient to estimate EE over a year-long period (52). To date, the scientific literature deeply studying alternative workstations is still novel and fragmentary. About this, Figure 2 summarizes the pros and cons of new alternative workstations to point out their strengths and shortcomings. In conclusion, alternative workstations are ideally relevant opportunities for acting on the reduced EE related to sedentary works. However, proposals of NEAT approaches in the workplace must be optimized in compliance with worker's devices acceptance, and the safeguard of the working tasks.

**Table 1:** Summary of energy expenditure (kcal/min and Kcal/yr) derived from the employment of alternative workstations (i.e., seated, standing, seated pedal, and walking).

**References**	**Workstation**	**EE (Kcal/min)**	**EE (Kcal/Yr)**	**Kg/Yr**
Reiff et al. (107)	Seated	1.02	118483.20	16.93
Speck et al. (53)		1.30	151008.00	21.57
Swartz et al. (108)		1.46	169593.60	24.23
Carr et al. (86)		0.99	114998.40	16.43
Koepp et al. (84)		1.35	156816.00	22.40
Horswill et al. (45)		1.43	166108.80	23.73
Mean		1.26	146168.00	20.88
SD		0.20	23753.70	3.39
Reiff et al. (107)	Standing	1.36	157977.60	22.57
Straker et al. (74)		1.36	157977.60	22.57
Speck et al. (53)		1.29	149846.40	21.41
Cox et al. (109)		1.08	125452.80	17.92
Horswill et al. (45)		1.54	178886.40	25.56
Mean		1.33	154028.16	22.00
SD		0.17	19255.95	2.75
Carr et al. (86)	Seated Pedal	2.14	248582.40	35.51
Koepp et al. (84)		1.60	185856.00	26.55
Horswill et al. (45)		1.65	191664.00	27.38
Mean		1.80	208700.80	29.81
SD		0.30	34660.35	4.95
Levine et al. (75)	Walking	1.96	227673.60	32.52
Koepp et al. (71)		2.90	336864.00	48.12
Koepp et al. (84)		2.80	325248.00	46.46
Mean		2.55	296595.20	42.37
SD		0.52	59969.77	8.57

Moreover, considering that 1 kg of fat is approximately equivalent to 7,000 kcals, the kilograms of fat consumed over a year-long period are also reported.

## Author contributions

AR, GM, and AP contributed to the literature review and classification. AR and AP wrote the first draft of the manuscript. GM and AP contributed to the manuscript revision and approved the submitted version. All authors contributed to the article and approved the submitted version.

## Conflict of interest

The authors declare that the research was conducted in the absence of any commercial or financial relationships that could be construed as a potential conflict of interest. The reviewer AB declared a past collaboration with the authors to the handling editor.

## Publisher's note

All claims expressed in this article are solely those of the authors and do not necessarily represent those of their affiliated organizations, or those of the publisher, the editors and the reviewers. Any product that may be evaluated in this article, or claim that may be made by its manufacturer, is not guaranteed or endorsed by the publisher.
